# Supplementary material for: Structural basis for assembly and disassembly of the IGF/IGFBP/ALS ternary complex
Source: Nat Commun. 2022 Jul 30;13:4434. doi: 10.1038/s41467-022-32214-2 (PMC9338993; doi:10.1038/s41467-022-32214-2)
Supplement: Supplementary file 1 — Supplementary Information [file 41467_2022_32214_MOESM1_ESM.pdf]

Supplementary Information

Structural basis of assembly and disassembly of the IGF/IGFBP/ALS ternary complex

Hyojin Kim, Yaoyao Fu, Ho Jeong Hong, Seong-Gyu Lee, Dong Sun Lee, Ho Min Kim

Supplementary Figures 1–7 and Legends

Supplementary Tables 1–6

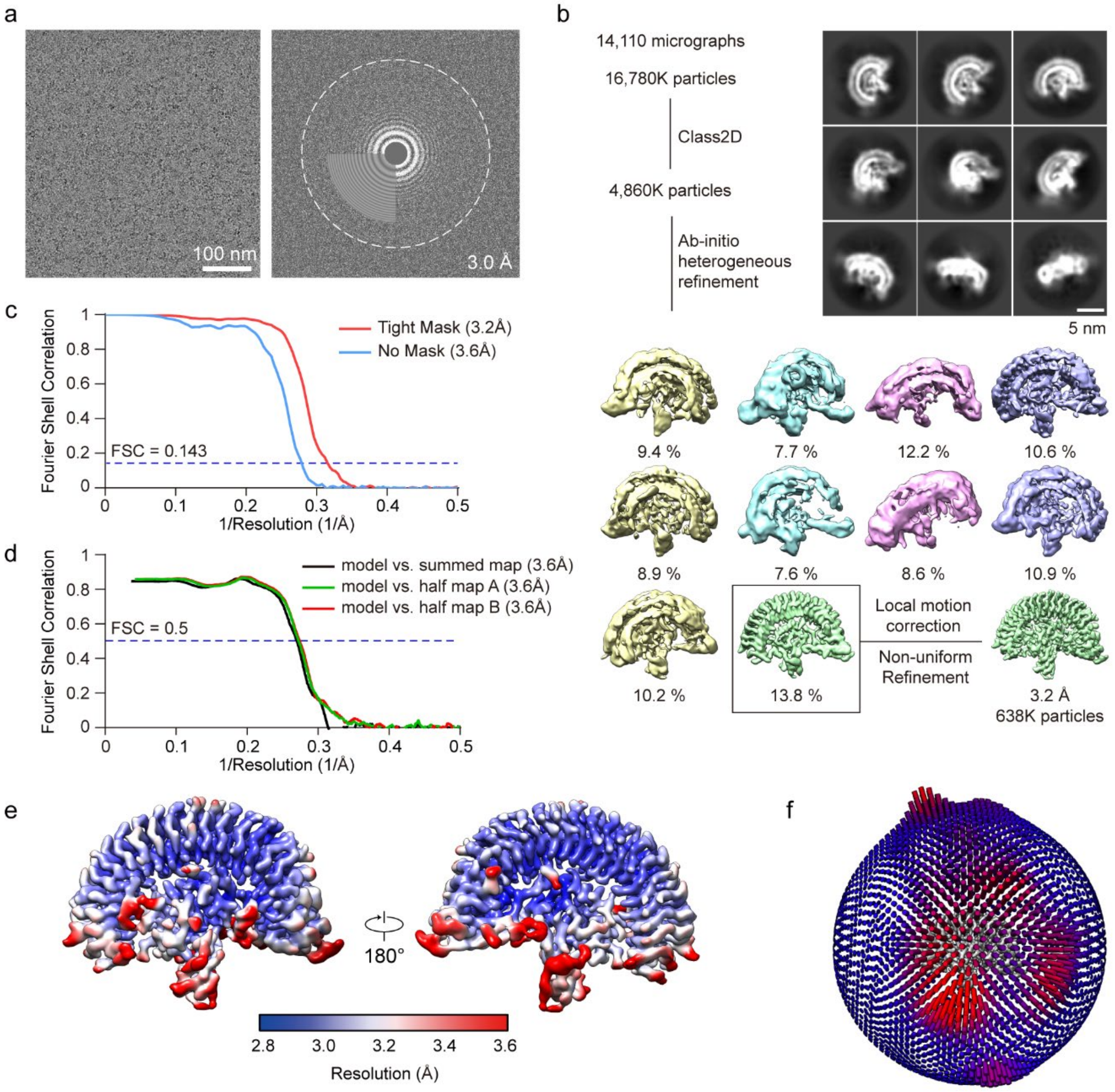

Supplementary Fig. 1 | Cryo-EM analysis of IGF1/IGFBP3/ALS ternary complex.

a, Representative cryo-EM micrograph (left) and its Fourier transform (right) among 14,110 micrographs.

b, Data processing workflow of cryo-EM analysis and representative 2D class averages of the IGF1/IGFBP3/ALS ternary complex.

c, Gold-standard Fourier shell correlation (FSC) between two independently refined half maps in cryoSPARC (resolution cutoff at FSC = 0.143).

d, FSC curves for cross-validation: model versus summed map (black), model versus half map A (used in test refinement, green), model versus half map 2 (not used in test refinement, red).

e, Final cryo-EM map colored with local resolution.

f, Euler angle distribution of all particles used in the final 3D reconstructions. The height and color (from blue to red) of the cylinder bars are proportional to the number of particles in those views.

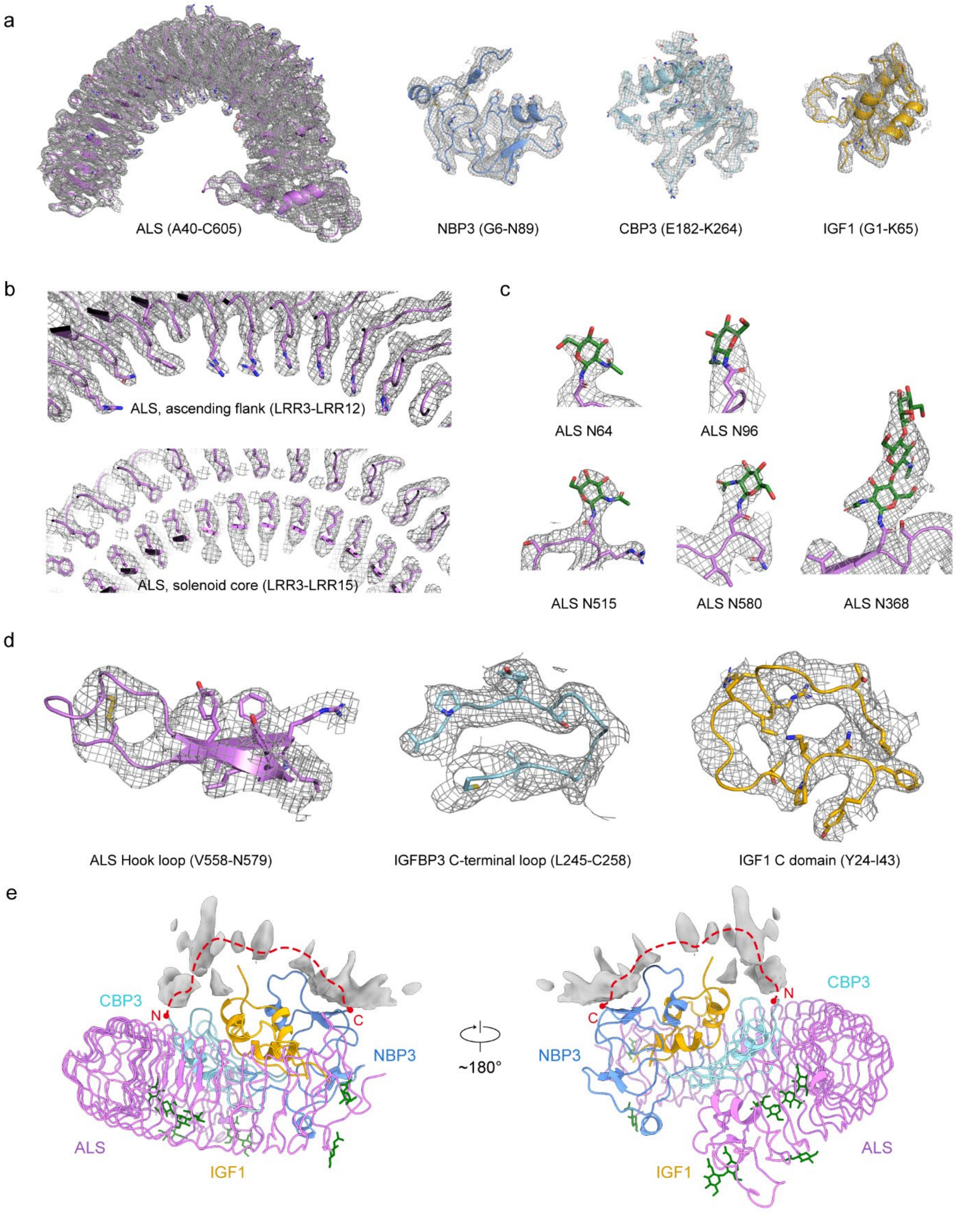

Supplementary Fig. 2 | Cryo-EM map for IGF1/IGFBP3/ALS complex.

a, Cryo-EM map and model of ALS (residues 40-605, contour level = 9), NBP3 (residues 6-89, contour level = 11), CBP3 (residues 182-264, contour level = 9), IGF1 (residues 1-65, contour level = 9).

b, Cryo-EM map and model of ascending flank (top, LRR3-12) and solenoid core (bottom, LRR3-15) of ALS (contour level = 13).

c, Cryo-EM map and model of glycosylation (N64, N96, N368, N580, and N515) of ALS (contour level = 9).

d, Cryo-EM map and model of ALS hook loop (residues 558-579, contour level = 8.5), IGFBP3 C-terminal loop (residues 245-258, contour level = 8.5), and IGF1 C domain (residues 24-43, contour level = 8.5).

e, Cryo-EM density for central linker domain of IGFBP3 (surface level = 0.088). C-terminal end of NBP3 and N-terminal end of CBP3 are marked with red dots, and the potential path for central linker domain of IGFBP3 is indicated with dotted red line.

a

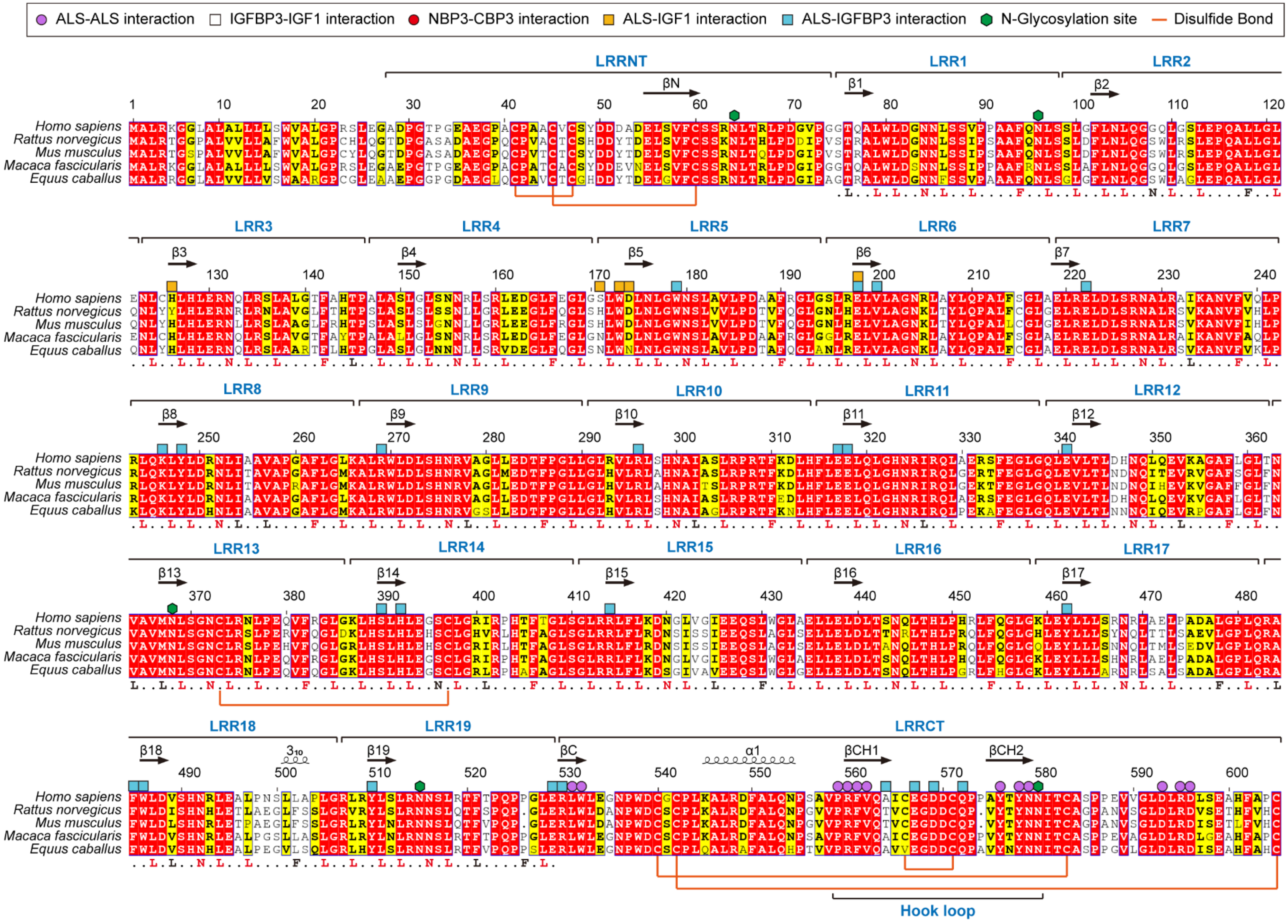
ALS-ALS interaction
IGFBP3-IGF1 interaction
NBP3-CBP3 interaction
ALS-IGF1 interaction
ALS-IGFBP3 interaction
N-Glycosylation site
Disulfide Bond
LRRNT
LRR1
LRR2
LRR3
LRR4
LRR5
LRR6
LRR7
LRR8
LRR9
LRR10
LRR11
LRR12
LRR13
LRR14
LRR15
LRR16
LRR17
LRR18
LRR19
LRRCT
Homo sapiens
Rattus norvegicus
Mus musculus
Macaca fascicularis
Equus caballus
Hook loop

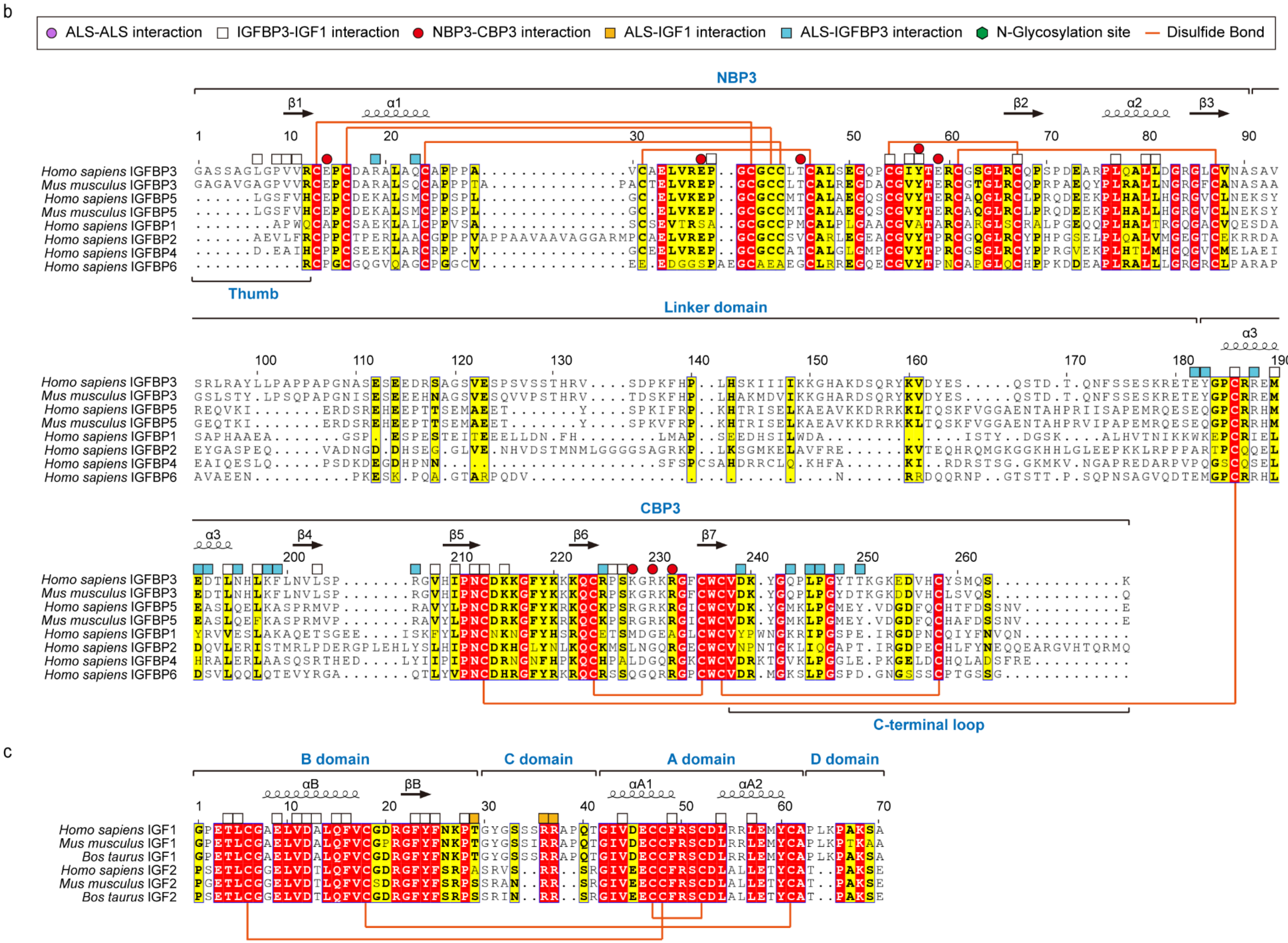

b
ALS-ALS interaction
IGFBP3-IGF1 interaction
NBP3-CBP3 interaction
ALS-IGF1 interaction
ALS-IGFBP3 interaction
N-Glycosylation site
Disulfide Bond
NBP3
Thumb
Linker domain
CBP3
C-terminal loop
Homo sapiens IGFBP3
Mus musculus IGFBP3
Homo sapiens IGFBP5
Mus musculus IGFBP5
Homo sapiens IGFBP1
Homo sapiens IGFBP2
Homo sapiens IGFBP4
Homo sapiens IGFBP6
c
B domain
C domain
A domain
D domain
αB
βB
αA1
αA2
Homo sapiens IGF1
Mus musculus IGF1
Bos taurus IGF1
Homo sapiens IGF2
Mus musculus IGF2
Bos taurus IGF2

Supplementary Fig. 3 | Multiple sequence alignment of ALS, IGFBPs, and IGFs.

a, Amino acid sequence alignment of human (*H. sapiens,* UniProt: P35858), rat (*R. norvegicus,* UniProt: P35859), mouse (*M. musculus,* UniProt: P70389), monkey (*P. hamadryas,* UniProt: O02833), and horse (*Equus caballus,* UniProt: F1P8U6) ALS. The LRR consensus sequence of ALS (xLxxLxLxxNxLxxLxxxxFxxLx) is labeled under the LRR motif alignment, and the conserved residues in the corresponding LRR motif are colored in red.

b, Amino acid sequence alignment of human IGFBP1-6 (*H. sapiens,* UniProt: P08833, P18065, P17936, P22692, P24593, and P24592) and mouse IGFBP3 and IGFBP 5 (*M. musculus,* UniProt: P47878 and Q07079).

c, Amino acid sequence alignment of human, mouse, and bovine IGF1 and IGF2 (*Homo sapiens,* UniProt: P05019 and P01344; *Mus musculus,* UniProt: P05017and P09535; and *Bos taurus,* UniProt: P07455 and P07456). α helix (A8-V17) and β strand (G22-F25) of IGF1 B domain, α1 helix (I43-F49) and α2 helix (L54-Y60) of IGF1 A domain are labeled as αB, βB, αA1, αA2, respectively.

(**a-c**) Domains (LRRNT, LRR1~LRR19 motif, LRRCT, and for ALS; NBP3, linker domain, CBP3 for IGFBP3; B, C, A, and D domain for IGF1) and secondary structure (arrows for β stands and helices for α-helices) elements are noted above the alignment. Additional domain regions (hook loop for ALS; thumb for IGFBP3) are marked under the alignment. Purple circles indicate intramolecular interacting residues in ALS. White squares indicate interacting residues between IGF1 and IGFBP3. Red circles indicate residues for interaction between NBP3 and CBP3. Yellow squares indicate interacting residues at the ALS and IGF1 interface. Cyan squares indicate interacting residues between ALS and IGFBP3. N-glycosylation site (green hexagon) and disulfide bond (orange line) are also indicated. Red boxes indicate perfect sequence conservation, whereas yellow boxes show residues with >70% similarity based on physicochemical properties. The sequence alignment was created using T-Coffee (http://tcoffee.crg.cat) and ESPript servers (http://espript.ibcp.fr).

a

ALS residues	ALS residues	Interaction
R560	D593	Ionic
	D596	Ionic
R595		Ionic
	N579	Hydrogen
L531	N579	Hydrogen
Y576	F561	Hydrophobic
Y578		Hydrophobic
P559	V562	Hydrophobic
W532		Hydrophobic

b

BP3 residues (Cleft)	IGF1 residues	Interaction
L77	L57	Hydrophobic
L80	L54	Hydrophobic
L81	L5	Hydrophobic
I56	A13	Hydrophobic
C67	F16	Hydrophobic
C54		Hydrophobic
P38		Hydrophobic
V208		Hydrophobic
P226	V11	Hydrophobic
I210		Hydrophobic
L194	V44	Hydrophobic
M190		Hydrophobic
C186	F49	Hydrophobic
C213		Hydrophobic
N212	E9	Hydrogen
K215	T4	Hydrogen
S227	Q15	Hydrogen
Y57	D12	Hydrogen
S227		Hydrogen

BP3 residues (Thumb)	IGF1 residues	Interaction
P9		Hydrophobic
L197	F25	Hydrophobic
L203		Hydrophobic
V11	F23	Hydrophobic
V208		Hydrophobic
L7	Y24	Hydrophobic
V10	P28	Hydrophobic

c

NBP3 residues	CBP3 residues	Interaction
E37		Ionic
T45	R230	Hydrogen
E59		Ionic
Y57	R232	Hydrophobic
E14	K228	Ionic

d

ALS residues	IGF1 residues	Interaction
H126	T29	Hydrogen
S171	R37	Hydrogen
W173		Hydrophobic
		Hydrophobic
D174	R36	Ionic
E198		Ionic

e

ALS residues (Concave)	BP3 residues (CBP3 helix)	Interaction
W179	F199	Hydrophobic
V200		Hydrophobic
E198	K198	Ionic
E222		Ionic
K246	E191	Ionic
Y248	N195	Hydrogen
R269	Y183	Hydrogen
R296	D192	Ionic
	E182	Hydrogen
E317		Ionic
E318	R188	Ionic
E341		Ionic

ALS residues (Concave)	BP3 residues (CBP3 loop)	Interaction
S390		Hydrogen
H392	Q243	Ionic
NAG_{368}		Hydrogen
R414	D239	Ionic
Y462	L245	Hydrophobic
W486	P246	Hydrophobic
Y510	Y248	Hydrophobic
E529		Hydrogen
R530	T249	Hydrogen

ALS residues (Hook loop)	BP3 residues	Interaction
E567	R225	Ionic
D569	R206	Ionic
Q572	R19	Ionic
	Q23	Ionic

Supplementary Fig. 4 | Interactions among IGF1, IGFBP3, and ALS.

a, Key amino acid residues of ALS involved in ALS hook loop stabilization.

b, Key amino acid residues involved in IGFBP3–IGF1 interactions.

c, Key amino acid residues of IGFBP3 involved in NBP3–CBP3 interactions.

d, Key amino acid residues involved in ALS–IGF1 interactions.

e, Key amino acid residues involved in ALS–IGFBP3 interactions.

(**a-e**) Interaction properties are indicated in red (ionic interaction), black (hydrogen bond), and blue (hydrophobic interaction). The residues participating in the main chain interaction are underlined.

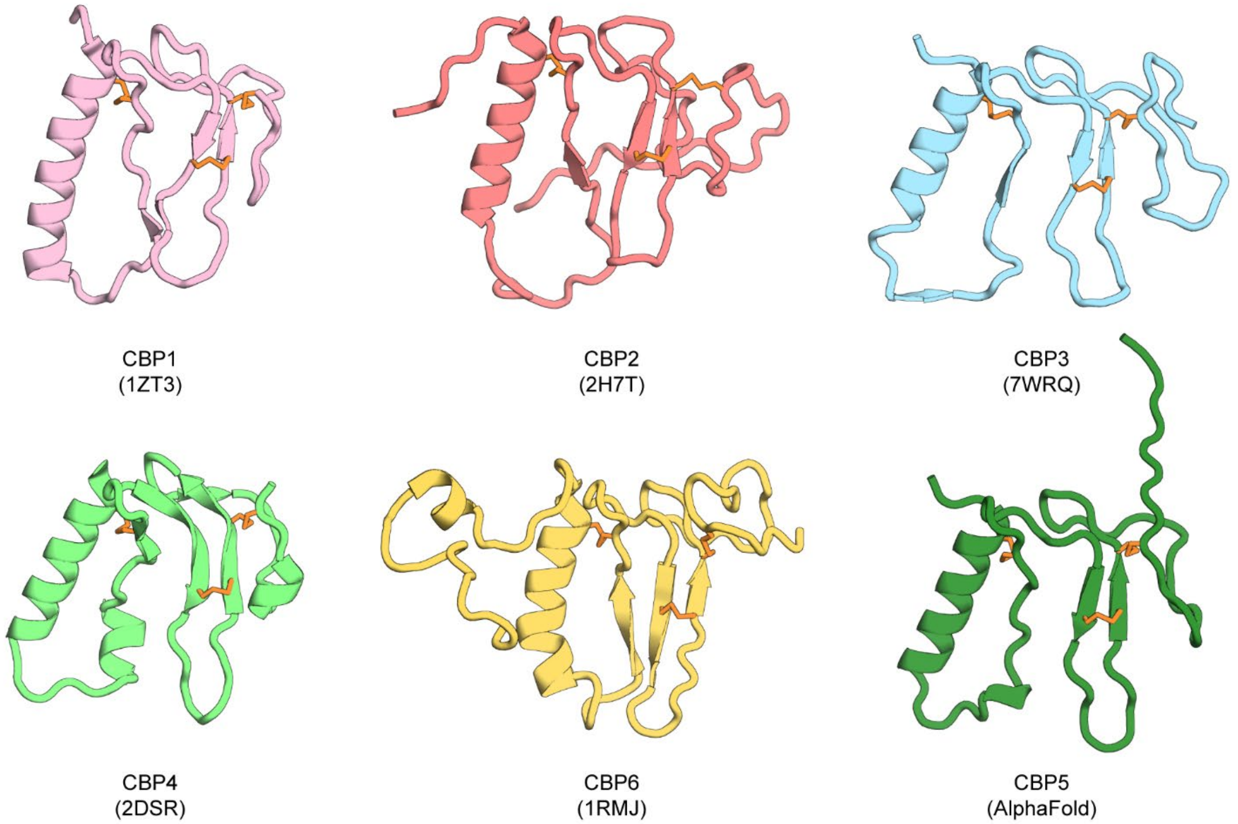

Cα rmsd	CBP1<br>(1ZT3)	CBP2<br>(2H7T)	CBP4<br>(2DSR)	CBP6<br>(1RMJ)
CBP3<br>(7WRQ)	2.040	3.118	1.132	3.789

Supplementary Fig. 5 | Structural comparison of IGFBP C-terminal domains.

Structure of each C-terminal domain (CBP) in IGFBP1 (PDB ID: 1ZT3, blue), IGFBP2 (PDB ID: 2H7T, deep salmon), IGFBP3 (PDB ID: 7WRQ, cyan), IGFBP4 (PDB ID: 2DSR, lime), IGFBP6 (PDB ID: 1RMJ, yellow), and IGFBP5 (AlphaFold, forest). CBP1, CBP3, and CBP4 are from the structure of the IGF1/IGFBP complexes, whereas CBP2 and CBP6 are structures of IGFBP only. CBP5 is a structure predicted by AlphaFold (UniProt: P24593). Disulfide bridges (orange) are shown as sticks. Calculated Cα root mean square deviations of each domain are listed in the bottom table.

a

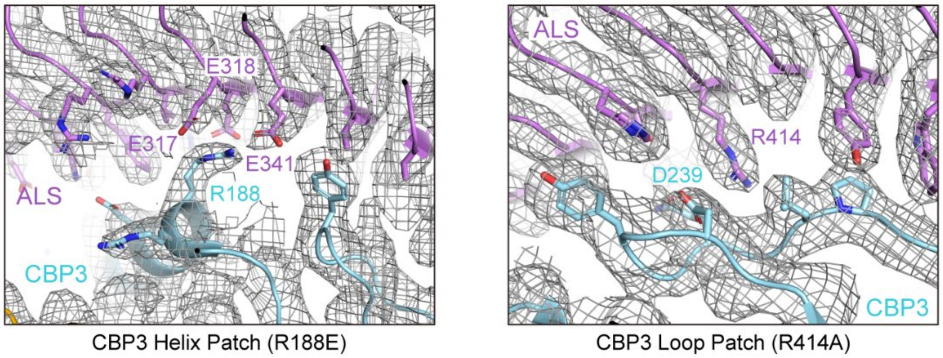

b

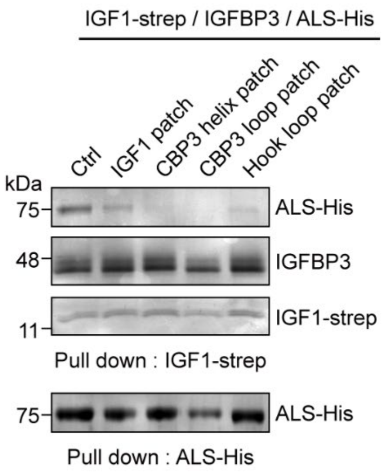

	Ctrl	IGF1 patch	CBP3 helix patch	CBP3 loop patch	Hook loop patch
ALS-His	wt	W173A	wt	R414A	Hook loop del
IGFBP3	wt	wt	R188E	wt	wt
IGF1-strep	wt	R36E/R37E	wt	wt	wt

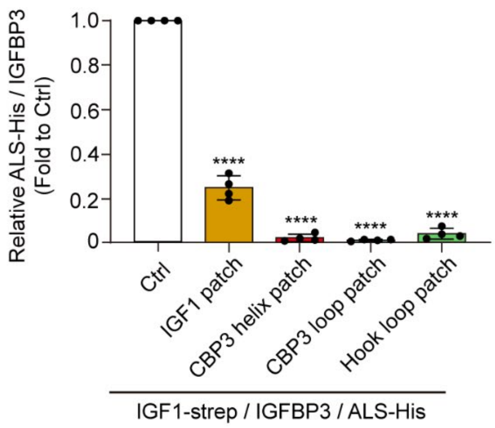

Supplementary Fig. 6 | Effects on ternary complex formation by mutations of key residues at the interface between ALS and the binary complex

a, Cryo-EM map and model of ALS and CBP3 in CBP3 helix patch (left, contour level = 8.5) and CBP3 loop patch (right, contour level = 9). Key residues for mutation and interacting residues are labeled.

b, SDS-PAGE for pull-down experiments with Expi 293F culture media co-expressing IGF1-strep, IGFBP3, and ALS-His WT or indicated mutants (left) and quantification of ALS bound to these binary complexes (right, bottom). The mutated key interacting residues at each patch and the construct combination for co-expression are indicated in the table (right, top). The components in the resulting complex were monitored by Coomassie staining after pull-down of IGF1-strep with Strep-Tactin resin. Expression of ALS mutants-His (W173A, R414A, and hook loop deletion) was confirmed by pull-down with Ni-NTA resin. Densitometric analyses of ALS-His/IGFBP3 ratios are shown (right, bottom). Data from four independent experiments ($n = 4$) were analyzed and expressed as mean ± SD (****$P < 0.0001$ vs. control). P values by one-way ANOVA test followed by Sidak's multiple comparisons test.

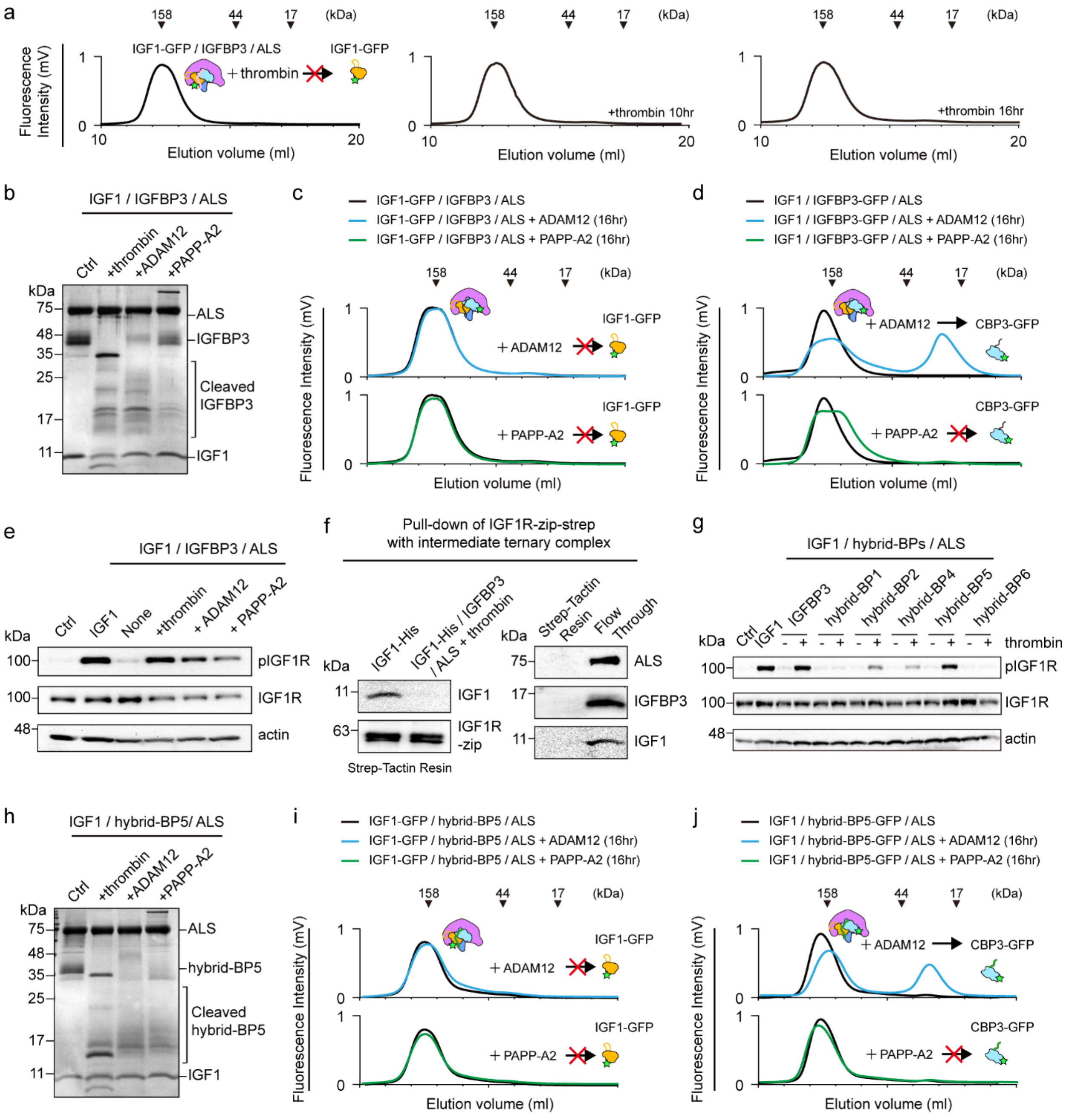

Supplementary Fig. 7 | Release of CBP3 from the ternary complex by thrombin and ADAM12 proteolysis, not by PAPP-A2 proteolysis.

a, FSEC of the ternary complex (IGF1-GFP/IGFBP3/ALS-His) after thrombin digestion for the indicated time (0.2 unit thrombin/1 µg ternary complex for 0, 10 and 16h). Fluorescence signal (GFP) was monitored to examine the dissociation of IGF1 from the ternary complex.

b, SDS-PAGE analysis of the IGF1-His/IGFBP3/ALS ternary complex after proteolysis (0.2 unit thrombin/1 µg ternary complex for 10 h; 0.2 µg ADAM12/1 µg ternary complex for 16 h; 0.2 µg

PAPP-A2/1 μg ternary complex for 16 h). IGF1-His/IGFBP3/ALS ternary complex was used for control (Ctrl).

c and d, FSEC of the ternary complex IGF1-GFP/IGFBP3/ALS-His (**c**), or IGF1/IGFBP3-GFP/ALS-His (**d**) after digestion with ADAM12 (Cyan, 0.2 μg ADAM12/1 μg ternary complex for 16 h) or PAPP-A2 (Green, 0.2 μg PAPP-A2/1 μg ternary complex for 16 h). Fluorescence signal (GFP) was monitored to examine the dissociation of IGF1 (**c**) or CBP3 (**d**) from the indicated ternary complex.

e, Immunoblot analysis for IGF1R phosphorylation after treatment of the intermediate ternary complex. HEK293A cells were treated with IGF1-His (5 nM, positive control), IGF1-His /IGFBP3/ALS ternary complex (5 nM, none), or intermediate ternary complex (5 nM). The intermediate ternary complex was prepared by protease digestion (0.2 unit thrombin/1 μg ternary complex for 10 h; 0.2 μg ADAM12/1 μg ternary complex for 16 h; 0.2 μg PAPP-A2/1 μg ternary complex for 16 h) and subsequent SEC purification. No treatment was used for negative control (Ctrl).

f, Pull-down of the IGF1R ectodomain-leucine zipper-Strep (IGF1R-zip-strep) with Strep-Tactin resin after mixing IGF1R-zip-strep with IGF1-His or the intermediate ternary complex (IGF1-His/IGFBP3/ALS ternary complex after thrombin proteolysis). Immunoblot analysis was performed with anti-His and anti-Strep antibody to detect IGF1 bound to IGF1R-zip-strep and IGR1R-zip-strep bound to Strep-Tactin resin, respectively (left). Immunoblot analysis was also performed with anti-ALS, anti-IGFBP3, anti-His antibody to detect ALS, IGFBP3, and IGF-His which are bound to IGF1R-zip-strep or are contained in flow through (right).

g, Immunoblot analysis for IGF1R phosphorylation with intermediate ternary complex containing hybrid-BPs. IGF1-His (5 nM, positive control), IGF1-His/IGFBP3/ALS ternary complexes (5 nM), or IGF1-His/hybrid-BPs/ALS ternary complexes (5 nM) with and without thrombin digestion (0.2 unit thrombin/1 μg ternary complex for 10 h) were treated onto HEK293A cells. No treatment for negative control (Ctrl).

h, SDS-PAGE analysis of the IGF1-His/hybrid-BP5/ALS ternary complex after proteolysis (0.2 unit thrombin/1 μg ternary complex for 10 h; 0.2 μg ADAM12/1 μg ternary complex for 16 h; 0.2 μg PAPP-A2/1 μg ternary complex for 16 h). The IGF1/hybrid-BP5/ALS ternary complex was used for control (Ctrl).

i and j, FSEC of the ternary complex IGF1-GFP/hybrid-BP5/ALS-His (**i**), or IGF1/hybrid-BP5-GFP/ALS-His (**j**) after digestion with ADAM12 (Cyan, 0.2 μg ADAM12/1 μg ternary complex for 16 h) or PAPP-A2 (Green, 0.2 μg PAPP-A2/1 μg ternary complex for 16 h). Fluorescence signal (GFP) was monitored to examine the dissociation of IGF1 (**i**) or CBP3 (**j**) from the indicated ternary complex.

(b, e, f, g and h) Similar results were observed in three independent experiments.

Supplementary Table 1. Cryo-EM data collection, refinement, and validation statistics

**Data collection and processing**	
Magnification	105,000
Voltage (kV)	300
Electron exposure ($e^-/Å^2$)	63.2
Defocus range (μm)	-0.9 ~ -2.7
Pixel size (Å)	0.883
Symmetry imposed	C1
Initial particle images (no.)	16,780,000
Final particle images (no.)	638,058
Map resolution (Å)	3.2
FSC threshold	0.143
**Refinement**	
Initial model used (PDB code)	2DSR
Map resolution (Å)	3.2
FSC threshold	0.143
Map sharpening B factor ($Å^2$)	0
Model composition	
Nonhydrogen atoms	6,247
Protein residues	798
Ligands	7
B factors ($Å^2$)	
Protein	130.19
Ligand	113.14
R.m.s. deviations	
Bond lengths (Å)	0.011
Bond angles (°)	1.274
**Validation**	
MolProbity score	1.55
Clashscore	0.96
Poor rotamers (%)	1.06
Ramachandran plot	
Favored (%)	79.62
Allowed (%)	19.87
Disallowed (%)	0.51

Supplementary Table 2. Acid labile subunit genes for Consurf analysis

	Acid labile subunit genes	
**Accession numbers**	NP_004961.1 (*Homo sapiens*)	XP_016081064.1 (*Ornithorhynchus anatinus*)
	NP_001351824.1 (*Mus musculus*)	XP_004705854.1 (*Echinops telfairi*)
	XP_038942850.1 (*Rattus norvegicus*)	XP_037013030.1 (*Artibeus jamaicensis*)
	NP_001069431.1 (*Bos taurus*)	XP_003916392.1 (*Papio anubis*)
	XP_003198203.1 (*Danio rerio*)	XP_004057012.2 (*Gorilla gorilla gorilla*)
	XP_001118634.1 (*Macaca mulatta*)	XP_025772696.1 (*Puma concolor*)
	XP_002940278.2 (*Xenopus tropicalis*)	XP_030728025.1 (*Globicephala melas*)
	XP_425222.3 (*Gallus gallus*)	XP_033695959.1 (*Tursiops truncatus*)
	XP_016784664.1 (*Pan troglodytes*)	XP_023472407.1 (*Equus caballus*)
	XP_038525519.1 (*Canis lupus familiaris*)	XP_010948208.1 (*Camelus bactrianus*)
	NP_001041651.1 (*Sus scrofa*)	XP_017896378.1 (*Capra hircus*)
	XP_005081648.1 (*Mesocricetus auratus*)	XP_027817538.1 (*Ovis aries*)
	XP_004750818.1 (*Mustela putorius furo*)	XP_017496939.2 (*Manis javanica*)
	XP_029433221.1 (*Rhinatrema bivittatum*)	XP_008120350.1 (*Anolis carolinensis*)
	XP_007906381.1 (*Callorhinchus milii*)	XP_009491144.1 (*Pelecanus crispus*)
	XP_027678915.1 (*Chelonia mydas*)	XP_032183150.1 (*Mustela erminea*)
	XP_034988039.1 (*Zootoca vivipara*)	XP_020862174.1 (*Phascolarctos cinereus*)
	XP_026538289.1 (*Notechis scutatus*)	XP_010565355.1 (*Haliaeetus leucocephalus*)
	XP_038043074.1 (*Anas platyrhynchos*)	XP_032701926.1 (*Lontra canadensis*)
	XP_035195408.1 (*Oxyura jamaicensis*)	XP_026714531.1 (*Athene cunicularia*)
	XP_021147646.1 (*Columba livia*)	XP_032053301.1 (*Aythya fuligula*)

Supplementary Table 3. Insulin-like growth factor-binding protein 3 genes for Consurf analysis

	Insulin-like growth factor-binding protein 3 genes	
**Accession numbers**	NP_000589.2 (*Homo sapiens*)	NP_001301148.1 (*Capra hircus*)
	NP_036720.2 (*Rattus norvegicus*)	XP_021503913.1 (*Meriones unguiculatus*)
	NP_032369.2 (*Mus musculus*)	XP_004630408.1 (*Octodon degus*)
	NP_776981.1 (*Bos taurus*)	XP_027692998.1 (*Vombatus ursinus*)
	NP_001005156.1 (*Sus scrofa*)	XP_012618881.1 (*Microcebus murinus*)
	XP_003639605.2 (*Canis lupus familiaris*)	XP_023373655.1 (*Otolemur garnettii*)
	NP_001152748.1 (*Ovis aries*)	XP_029090218.1 (*Monodon monoceros*)
	NP_001247476.1 (*Macaca mulatta*)	XP_020032653.1 (*Castor canadensis*)
	XP_519084.2 (*Pan troglodytes*)	XP_036859050.1 (*Manis javanica*)
	XP_023494800.1 (*Equus caballus*)	XP_026357817.1 (*Ursus arctos horribilis*)
	XP_027307091.2 (*Anas platyrhynchos*)	XP_025289672.1 (*Canis lupus dingo*)
	XP_006051369.2 (*Bubalus bubalis*)	XP_023082252.1 (*Piliocolobus tephrosceles*)
	XP_008840408.1 (*Nannospalax galili*)	XP_017380055.1 (*Cebus imitator*)
	XP_011760841.1 (*Macaca nemestrina*)	XP_028633334.1 (*Grammomys surdaster*)
	XP_003896056.1 (*Papio anubis*)	XP_032614032.1 (*Hylobates moloch*)
	XP_025234541.1 (*Theropithecus gelada*)	XP_010372901.1 (*Rhinopithecus roxellana*)
	XP_009241083.1 (*Pongo abelii*)	XP_037349128.1 (*Talpa occidentalis*)
	XP_027430511.1 (*Zalophus californianus*)	XP_005319317.1 (*Ictidomys tridecemlineatus*)
	XP_004283046.1 (*Orcinus orca*)	XP_040500426.1 (*Ursus maritimus*)
	XP_003407178.1 (*Loxodonta africana*)	XP_023970916.1 (*Physeter catodon*)
	XP_020771079.1 (*Odocoileus virginianus texanus*)	XP_026979864.1 (*Lagenorhynchus obliquidens*)

Supplementary Table 4. Insulin-like growth factor 1 genes for Consurf analysis

	Insulin-like growth factor 1 genes	
**Accession numbers**	NP_000609.1 (*Homo sapiens*)	XP_006141463.1 (*Tupaia chinensis*)
	NP_001104745.1 (*Mus musculus*)	XP_029455549.1 (*Rhinatrema bivittatum*)
	NP_001004384.1 (*Gallus gallus*)	XP_007072330.1 (*Chelonia mydas*)
	XP_012015032.1 (*Ovis aries*)	XP_005235639.1 (*Falco peregrinus*)
	XP_005206554.1 (*Bos taurus*)	XP_021243075.1 (*Numida meleagris*)
	XP_005664255.1 (*Sus scrofa*)	XP_009092444.1 (*Serinus canaria*)
	NP_001300784.1 (*Canis lupus familiaris*)	XP_015483546.1 (*Parus major*)
	NP_001075967.2 (*Equus caballus*)	XP_009647544.1 (*Egretta garzetta*)
	XP_519084.2 (*Pan troglodytes*)	XP_023792657.1 (*Cyanistes caeruleus*)
	XP_023494800.1 (*Equus caballus*)	XP_011760043.1 (*Macaca nemestrina*)
	NP_001247655.1 (*Macaca mulatta*)	XP_002915500.1 (*Ailuropoda melanoleuca*)
	XP_005022610.1 (*Anas platyrhynchos*)	XP_002199849.1 (*Taeniopygia guttata*)
	XP_002936875.1 (*Xenopus tropicalis*)	XP_007130866.1 (*Physeter catodon*)
	XP_006058875.1 (*Bubalus bubalis*)	XP_010598061.1 (*Loxodonta africana*)
	XP_005303753.1 (*Chrysemys picta bellii*)	XP_010988323.1 (*Camelus dromedarius*)
	XP_003907099.1 (*Papio anubis*)	XP_017536871.1 (*Manis javanica*)
	XP_005482778.1 (*Zonotrichia albicollis*)	XP_026253508.1 (*Urocitellus parryii*)
	XP_022455544.1 (*Delphinapterus leucas*)	XP_008142970.1 (*Eptesicus fuscus*)
	XP_003989226.1 (*Felis catus*)	XP_006205960.1 (*Vicugna pacos*)
	XP_005500337.1 (*Columba livia*)	XP_021531233.1 (*Aotus nancymaae*)
	XP_006186104.1 (*Camelus ferus*)	NP_001273775.1 (*Alligator sinensis*)
	XP_005421104.1 (*Geospiza fortis*)	XP_027536273.1 (*Neopelma chrysocephalum*)

Supplementary Table 5. Constructs for recombinant protein expression

Construct	Expression vector	Information
IGF1	pcDNA3.1	HindIII-IGF1(M1-A118)-stop-XhoI
IGF1-proA	pcDNA3.1	HindIII-IGF1(M1-A118)-thrombin site-Protein A tag-XhoI
IGF1-His	pcDNA3.1	HindIII-IGF1(M1-A118)-BamHI-6x His tag-XbaI
IGF1-Strep	pcDNA3.1	HindIII-IGF1(M1-A118)-Twin-Strep-tag-XhoI
IGF1-GFP-Strep	pcDNA3.1	HindIII-IGF1(M1-A118)-GGGGS linker-EGFP-Twin-Strep-tag-XhoI
IGF1(R36E/R37E)-Strep	pcDNA3.1	HindIII-IGF1(M1-A118, R36E/R37E)-Twin-Strep-tag-XhoI
IGFBP3	pcDNA3.1	Signal peptide-BamHI-IGFBP3(G28-K291)-stop-XhoI
IGFBP3-Strep	pcDNA3.1	Signal peptide-BamHI-IGFBP3(G28-K291)-Twin-Strep-tag-XhoI
Myc-IGFBP3-Strep	pcDNA3.1	Signal peptide-BamHI-Myc tag-IGFBP3(G28-K291)-NotI-Twin-Strep-tag-XhoI
IGFBP3-GFP-Strep	pcDNA3.1	Signal peptide-BamHI-IGFBP3(G28-K291)-$(GGGGS)_2$ linker-EGFP-Twin-Strep-tag-XhoI
CLD3-CBP3-Strep	pcDNA3.1	Signal peptide-BamHI-IGFBP3(S118-K291)-Twin-Strep-tag-XhoI
IGFBP3(R188E)	pcDNA3.1	Signal peptide-BamHI-IGFBP3(G28-K291, R188E)-stop-XhoI
Hybrid-BP1-Strep	pcDNA3.4 TOPO	Signal peptide-BamHI-NBP3(G28-A118)-CLD1(S108-W172)-CBP3(Y210-K291)-Twin-Strep-tag-XhoI
Hybrid-BP2-Strep	pcDNA3.4 TOPO	Signal peptide-BamHI-NBP3(G28-A118)-CLD2(R135-A223)-CBP3(Y210-K291)-Twin-Strep-tag-XhoI
Hybrid-BP4-Strep	pcDNA3.4 TOPO	Signal peptide-BamHI-NBP3(G28-A118)-CLD4(A104-P170)-CBP3(Y210-K291)-Twin-Strep-tag-XhoI
Hybrid-BP5-Strep	pcDNA3.4 TOPO	Signal peptide-BamHI-NBP3(G28-A118)-CLD5(K104-E188)-CBP3(Y210-K291)-Twin-Strep-tag-XhoI
Hybrid-BP6-Strep	pcDNA3.4 TOPO	Signal peptide-BamHI-NBP3(G28-A118)-CLD6(R108-E159)-CBP3(Y210-K291)-Twin-Strep-tag-XhoI
Hybrid-BP1-GFP-Strep	pcDNA3.4 TOPO	Signal peptide-BamHI-NBP3(G28-A118)-CLD1(S108-W172)-CBP3(Y210-K291)-$(GGGGS)_2$ linker-EGFP-Twin-Strep-tag-XhoI
Hybrid-BP2- GFP-Strep	pcDNA3.4 TOPO	Signal peptide-BamHI-NBP3(G28-A118)-CLD2(R135-A223)-CBP3(Y210-K291)-$(GGGGS)_2$ linker-EGFP-Twin-Strep-tag-XhoI
Hybrid-BP4- GFP-Strep	pcDNA3.4 TOPO	Signal peptide-BamHI-NBP3(G28-A118)-CLD4(A104-P170)-CBP3(Y210-K291)-$(GGGGS)_2$ linker-EGFP-Twin-Strep-tag-XhoI
Hybrid-BP5- GFP-Strep	pcDNA3.4 TOPO	Signal peptide-BamHI-NBP3(G28-A118)-CLD5(K104-E188)-CBP3(Y210-K291)-$(GGGGS)_2$ linker-EGFP-Twin-Strep-tag-XhoI

Hybrid-BP6- GFP-Strep	pcDNA3.4 TOPO	Signal peptide-BamHI-NBP3(G28-A118)-CLD6(R108-E159)-CBP3(Y210-K291)-$(GGGGS)_2$ linker-EGFP-Twin-Strep-tag-XhoI
ALS	pcDNA3.1	Signal peptide-BamHI-ALS(A28-C605)-stop-XhoI
ALS-His	pcDNA3.1	Signal peptide-ALS(A28-C605)-BamHI-6x His tag-XbaI
ALS (W173A)-His	pcDNA3.1	Signal peptide- ALS(A28-C605, W173A)-BamHI-6x His tag-XhaI
ALS (R414A)-His	pcDNA3.1	Signal peptide- ALS(A28-C605, R414A)-BamHI-6x His tag-XhaI
ALS (hook loop del)-His	pcDNA3.1	Signal peptide- ALS(A28-560R, 579N-605C)-BamHI-6x His tag-XhaI
IGF1R-zip	pcDNA3.1	Signal peptide-BamHI-IGF1R ecto(E31-H935)-GCN4 zipper-NotI-Twin-Strep-tag-XhoI

Supplementary Table 6. Primer sequences used for cloning

Primer	Sequence (5'–3')
IGF1-F	TTC TCT CCA CAG AAG CTT ATG GGT AAG ATC AGC AGC CTT CCA A
IGF1-R	GCG GAA CCA GTC TAG ATC AGG CTG ATT TGG CAG GTT TGA
IGF1-proA-R	ACG CGG AAC CAG TCT AGA GGC TGA TTT GGC AGG TTT GA
IGF1-His-F	CCT TTC TCT CCA CAG AAG CTT ATG GGT AAG ATC AGC AGC C
IGF1-His-R	CAG TGA TGA TGA TGA TGA TGG GAT CCG GCT GAT TTG GCA GGT TTG
IGF1-Strep-R	GGA TGG CTC CAA GCG CTT GCG GCC GCG GCT GAT TTG GCA GGT TTG
IGF1-GFP-Strep-F	GCC AAA TCA GCC GGC GGT GGC GGC TCA ATG GTG AGC AAG GGC GAG GAG C
IGF1-GFP-Strep-R1	CTT GCT CAC CAT TGA GCC GCC ACC GCC GGC TGA TTT GGC AGG TTT GAG G
IGF1-GFP-Strep-R2	CAG AGG TTG ATT CTC GAG TTA CTT CTC AAA CTG TGG GTG TGA C
IGF1 (R36E/R37E)-Strep-F1	ACC GGG TAT GGC TCC TCA TCC GAG AGG GCG CCT CAG ACA GGC ATT
IGF1 (R36E/R37E)-Strep-R1	AAT GCC TGT CTG AGG CGC CCT CTC GGA TGA GGA GCC ATA CCC GGT
IGF1 (R36E/R37E)-Strep-F2	GCT CCT CAT CCG AGG CGG CGC CTC AGA CAG
IGF1 (R36E/R37E)-Strep-R2	CTG TCT GAG GCG CCG CCT CGG ATG AGG AGC
IGFBP3-F	CCT TTG CGG CGG ATC CCG GCG CGA GCT CGG CGG GC
IGFBP3-R	GCG GAA CCA GTC TCT ACT TGC TCT GCA TGC TGT AGC AGT G
IGFBP3-Strep-F	CAC AGG ATC TAG TTC AGG TGG ATC CGG CGC GAG CTC GGC GGG CTT G
IGFBP3-Strep-R	GGA TGG CTC CAA GCG CTT GCG GCC GCC TTG CTC TGC ATG CTG
Myc-IGFBP3-Strep-F1	CTC ATC TCA GAA GAG GAT CTG GGC GCG AGC TCG GCG GGC TTG GG
Myc-IGFBP3-Strep-F2	CTC ACA GGA TCT AGT TCA GGT GGA TCC GAA CAA AAA CTC ATC TCA GAA GAG G
Myc-IGFBP3-Strep-R	GGA TGG CTC CAA GCG CTT GCG GCC GCC TTG CTC TGC ATG CTG
IGFBP3-GFP-Strep-F1	GGC GGT TCA GGC GGA GGT GGC TCT ATG GTG AGC AAG GGC GAG GAG C
IGFBP3-GFP-Strep-F2	GCT ACA GCA TGC AGA GCA AGG CGG CCG CAG GTG GAG GCG GTT CAG GCG G
IGFBP3-GFP-Strep-R	TTT GTA ATC CAG AGG TTG ATT CTC GAG TTA CTT CTC AAA CTG TGG GTG
CLD3-CBP3-Strep-F	GGA TCT AGT TCA GGT GGA TCC AGT GCC GTC AGC CGC CTG CG

CLD3-CBP3-Strep-R	GAG GAT GGC TCC AAG CGC TTG CGG CCG CCT TGC TCT GCA TGC TGT AGC
IGFBP3 (R188E)-F	GAC AGA ATA TGG TCC CTG CCG TGA AGA AAT GGA AGA CAC AC
IGFBP3 (R188E)-R	GTG TGT CTT CCA TTT CTT CAC GGC AGG GAC CAT ATT CTG TC
Hybrid-BP1-F	CGC GGG CTC TGC GTC AAC GCT TCT GAC GCC TCC GCT CCC
Hybrid-BP1-R	CCA TTT CTC TAC GGC AGG GAC CAT ACC ATT TTT TGA TGT TGG TGA CAT GG
Hybrid-BP2-F	CGC GGG CTC TGC GTC AAC GCT CGG GAC GCC GAG TAT GGC
Hybrid-BP2-R	CCA TTT CTC TAC GGC AGG GAC CAT AGG CAG GGG GTG GTC GCA GC
Hybrid-BP4-F	CGC GGG CTC TGC GTC AAC GCT GCG GAG ATC GAG GCC ATC C
Hybrid-BP4-R	CCA TTT CTC TAC GGC AGG GAC CAT AGG GCA CAG GCC GGG CAT CCT CCC
Hybrid-BP5-F	CGC GGG CTC TGC GTC AAC GCT AAG AGC TAC CGC GAG CAA GTC AAG ATC G
Hybrid-BP5-R	CCA TTT CTC TAC GGC AGG GAC CAT ACT CAG ACT CCT GTC TCA TCT CAG G
Hybrid-BP6-F	CGC GGG CTC TGC GTC AAC GCT CGC GCG CCT GCT GTT GCA GAG G
Hybrid-BP6-R	CCA TTT CTC TAC GGC AGG GAC CAT ACT CAG TGT CTT GGA CAC CCG C
Hybrid-BP-GFP-Strep-F	GGA GGA CGT GCA CTG CTA CAG CAT GCA GAG CAA GGC GGC CGC
Hybrid-BP-GFP-Strep-R	CCG GTA GGG ATC GAA CCC TTC TCG AGT TAC TTC TCA AAC TGT GGG TGT GAC C
ALS-F	GCT CCC TGC GCG GCC GCA TGA CTG GTT CCG CGT GGT TC
ALS-R	GAA CCA CGC GGA ACC AGT CAT GCG GCC GCG CAG GGA GC
ALS-His-F	TTC TCT CCA CAG AAG CTT ATG GCT CTG AGG AAA GGA GGG CT
ALS-His-R	ACG CGG AAC CAG TCT AGA GCA GGG AGC AAA ATG GGC TTC CGA CAA
ALS (W173A)-His-F	GGT CTC GGC AGC CTC GCG GAT CTC AAT CTC GG
ALS (W173A)-His-R	CCG AGA TTG AGA TCC GCG AGG CTG CCG AGA CC
ALS (R414A)-His-F	GGT CTC CGC GCT CTC TTC CTC AAG GAT AAC GGC CTC GTG GGC A
ALS (R414A)-His-R	GAA GAG AGC GCG GAG ACC AGA GAG ACC GGT GAA GGT GTG
ALS (hook loop del)-His-F1	CCA CTT TGC CTT TCT CTC CAC AGA AGC TTA TGG CTC TGA GGA AAG GAG GGC
ALS (hook loop del)-His-R1	CCC ACG ACC TCG GGA GGG CTG GCA CAG GTG ATG TTG TTG CGG GGC ACA GCA CTG GGG
ALS (hook loop del)-His-F2	CCC CAG TGC TGT GCC CCG CAA CAA CAT CAC CTG TGC C

ALS (hook loop del)-His-R2	GTG ATG ATG ATG ATG ATG GGA TCC GCA GGG AGC AAA ATG GGC
IGF1R-zip-F1	CTC ACA GGA TCT AGT TCA GGT GGA TCC GAA ATC TGC GGG CCA GGC ATC
IGF1R-zip-R1	GAG GAT GGC TCC AAG CGC TTG CGG CCG CAT GGA TGA AGT TTT CAT ATC C
IGF1R-zip-F2	GGC CAA AAC AGG ATA TGA AAA CTT CAT CCA TCG TTC GAT TCC AC
IGF1R-zip-R2	CGA ACT GAG GAT GGC TCC AAG CGC TTG CGG CCG CG
