# Supplementary material for: Structural basis for assembly and disassembly of the IGF/IGFBP/ALS ternary complex
Source: Nat Commun. 2022 Jul 30;13:4434. doi: 10.1038/s41467-022-32214-2 (PMC9338993; doi:10.1038/s41467-022-32214-2)
Supplement: Supplementary file 3 — Reporting Summary [file 41467_2022_32214_MOESM3_ESM.pdf]

nature portfolio

Corresponding author(s): Ho Min Kim

Last updated by author(s): Jul 11, 2022

Reporting Summary

Nature Portfolio wishes to improve the reproducibility of the work that we publish. This form provides structure for consistency and transparency in reporting. For further information on Nature Portfolio policies, see our Editorial Policies and the Editorial Policy Checklist.

Statistics

For all statistical analyses, confirm that the following items are present in the figure legend, table legend, main text, or Methods section.

n/a	Confirmed	
☐	☒	The exact sample size (*n*) for each experimental group/condition, given as a discrete number and unit of measurement
☐	☒	A statement on whether measurements were taken from distinct samples or whether the same sample was measured repeatedly
☐	☒	The statistical test(s) used AND whether they are one- or two-sided<br>*Only common tests should be described solely by name; describe more complex techniques in the Methods section.*
☒	☐	A description of all covariates tested
☒	☐	A description of any assumptions or corrections, such as tests of normality and adjustment for multiple comparisons
☐	☒	A full description of the statistical parameters including central tendency (e.g. means) or other basic estimates (e.g. regression coefficient) AND variation (e.g. standard deviation) or associated estimates of uncertainty (e.g. confidence intervals)
☐	☒	For null hypothesis testing, the test statistic (e.g. *F*, *t*, *r*) with confidence intervals, effect sizes, degrees of freedom and *P* value noted<br>*Give P values as exact values whenever suitable.*
☒	☐	For Bayesian analysis, information on the choice of priors and Markov chain Monte Carlo settings
☒	☐	For hierarchical and complex designs, identification of the appropriate level for tests and full reporting of outcomes
☒	☐	Estimates of effect sizes (e.g. Cohen's *d*, Pearson's *r*), indicating how they were calculated

Our web collection on statistics for biologists contains articles on many of the points above.

Software and code

Policy information about availability of computer code

Data collection	Cryo-EM data was collected automatically using Thermo Scientific EPU v2.9 at Institute for Basic Science (IBS, Republic of Korea). Data collection statistics are shown in Supplementary Table 1.
Data analysis	CryoSPARC v3, MotionCor2, CTFFIND4 v4.1.10, Alphafold v2, DeepEMhancer, COOT v0.8.9.2 and PHENIX v1.19.2 were used for data processing, model building and structure refinement. All structure figures were generated using UCSF Chimera v.1.1.4, ChimeraX v.1.3, and PyMOL v2.3.1. GraphPad Prism v9.2.0 and ImageJ v.1.52 was used as well for data analysis. 3D structures were compared using DALI. Sequence alignment was created using T-Coffee and ESPript v.3.

For manuscripts utilizing custom algorithms or software that are central to the research but not yet described in published literature, software must be made available to editors and reviewers. We strongly encourage code deposition in a community repository (e.g. GitHub). See the Nature Portfolio guidelines for submitting code & software for further information.

Data

Policy information about availability of data

All manuscripts must include a data availability statement. This statement should provide the following information, where applicable:

- Accession codes, unique identifiers, or web links for publicly available datasets
- A description of any restrictions on data availability
- For clinical datasets or third party data, please ensure that the statement adheres to our policy

The cryo-EM density map of IGF1/IGFBP3/ALS complex has been deposited in the Electron Microscopy Data Bank (EMDB; https://www.ebi.ac.uk/pdbe/emdb/) under accession number EMD-32735. The atomic coordinates of IGF1/IGFBP3/ALS complex have been deposited in the Protein Data Bank (PDB; https://www.rcsb.org) with accession code 7WRQ. They will be available upon publication.

All the other data and codes used for the analysis are available from the corresponding author upon reasonable request.
The structural data from PDB used in this study are as follows : 2DSR (IGF1/NBP4/CBP4 complex), 2DSQ (IGF1/NBP4/CBP1 complex), 1H02 (free IGF1), 1WQJ (IGF1/NBP4 complex), 2L29 (IGF2/IGF2R complex), 1ZT3 (CBP of IGFBP1), 2H7T (CBP of IGFBP2), 1RMJ (CBP of IGFBP6).
The sequence data from UniProt used in sequence alignments are as follows : P35858 (human ALS), P35859 (rat ALS), P70389 (mouse ALS), O02833 (monkey ALS), F1P8U6 (horse ALS), P08833 (human IGFBP1), P18065 (human IGFBP2), P17936 (human IGFBP3), P22692 (human IGFBP4), P24593 (human IGFBP5), P24592 (human IGFBP6), P47878 (mouse IGFBP3), Q07079 (mouse IGFBP5), P05019 (human IGF1), P01344 (human IGF2), P05017 (mouse IGF1), P09535 (mouse IGF2), P07455 (cattle IGF1), and P07456 (cattle IGF2).
The gene data used in Consurf analysis are all listed in Supplementary Table 2-4.

Field-specific reporting

Please select the one below that is the best fit for your research. If you are not sure, read the appropriate sections before making your selection.

☒ Life sciences ☐ Behavioural & social sciences ☐ Ecological, evolutionary & environmental sciences

For a reference copy of the document with all sections, see nature.com/documents/nr-reporting-summary-flat.pdf

Life sciences study design

All studies must disclose on these points even when the disclosure is negative.

Sample size

For cryo-EM samples, twelve grids of the IGF1/IGFBP3/ALS ternary complex were pre-screened to identify the optimal grid for data collection. The amount of cryo-EM micrographs collected was based on the cryo-EM time allocation and previous knowledge estimating that the size is sufficient to generate a high-resolution density. The details of cryo-EM datasets including sample sizes are given in Methods and Supplementary Information. No sample size calculation was performed. Sample size was chosen by following the literature in the field (Li et al., Nature communications, 2019; Kavran et al., Elife, 2014).

Data exclusions

All acquired images were analyzed and parts were later excluded as is usual for such image processing studies. Misaligned image segments were excluded from averages based on cross-correlation scores and visual analysis (described in Methods section and Supplementary Figure 1).

Replication

Three independent samples were used for co-expression test (Fig. 5a, 5b, 5d, 5b, and Supplementary Fig. 6b), proteolysis test (Fig.6b, 6e, and Supplementary Fig. 7b, 7h), IGF1R pull-down test (Supplementary Fig. 6b), and immunoblot analysis (Fig. 6d and Supplementary Fig. 7e). Five independent samples were used for immunoblot analysis (Fig. 6f and Supplementary Fig. 7g). We repeated biochemical assays to confirm reproducibility, and similar results were obtained.

Randomization

For cryo-EM data collection, all particles were randomly distributed in the ice. For 3D refinement and resolution estimation, all particles were randomly split into two groups. Experiments involving cell biological analysis (immunoblot) were assured randomization through double-blind experiments. Randomization is not relevant for biochemical analysis (protein purification, fluorescence-detection size-exclusion chromatography and SDS-PAGE gel).

Blinding

The initial cryo-EM micrographs were manually picked to exclude bad images with thick ice or contamination. Blinding for biochemical analysis (protein purification, fluorescence-detection size-exclusion chromatography and SDS-PAGE gel) was not relevant as the reported data were not based on subjective judgment or interpretation, but quantitative measurements. The investigators were blinded to allocation during cell biological experiments and outcome analysis.

Reporting for specific materials, systems and methods

We require information from authors about some types of materials, experimental systems and methods used in many studies. Here, indicate whether each material, system or method listed is relevant to your study. If you are not sure if a list item applies to your research, read the appropriate section before selecting a response.

Materials & experimental systems

n/a	Involved in the study	
☐	☒	Antibodies
☐	☒	Eukaryotic cell lines
☒	☐	Palaeontology and archaeology
☒	☐	Animals and other organisms
☒	☐	Human research participants
☒	☐	Clinical data
☒	☐	Dual use research of concern

Methods

n/a	Involved in the study	
☒	☐	ChIP-seq
☒	☐	Flow cytometry
☒	☐	MRI-based neuroimaging

Antibodies

Antibodies used

1. Rabbit monoclonal anti-Myc-Tag (71D10) : Cell Signaling Technology, Cat# 2278 (1:1,000 for immunoblots)
2. Rabbit polyclonal anti-Strep tag II : abcam, Cat# ab76949 (1:1,000 for immunoblots)

3. Mouse monoclonal anti-His tag (6G2A9) : GenScript, Cat#A00186 (1:1,000 for immunoblots)
4. Rabbit monoclonal anti-IGFBP3 (D1U9C) : Cell Signaling Technology, Cat# 25864 (1:1,000 for immunoblots)
5. Mouse monoclonal anti-IGFBP3 (E-9) : Santa Cruz, Cat# sc-374365 (1:1,000 for immunoblots)
6. Mouse monoclonal anti-ALS (E-2) : Santa Cruz, Cat# sc-377131 (1:1,000 for immunoblots)
7. Mouse monoclonal anti-IGF1 (1C5-1A2) : Thermo Fisher, Cat# MA1-088 (1:1,000 for immunoblots)
8. Goat polyclonal anti-mouse IgG-HRP : Thermo Fisher, Cat# 62-6520 (1:5,000 for immunoblots)
9. Goat polyclonal anti-rabbit IgG-HRP : Thermo Fisher, Cat# 31460 (1:5,000 for immunoblots)
10. Rabbit monoclonal anti-Phospho-IGF1 Receptor beta (Tyr1135/1136) (19H7) : Cell Signaling Technology, Cat# 3024 (1:1,000 for immunoblots)
11. Rabbit ponoclonal anti-IGF1 Receptor beta : Cell Signaling Technology, Cat# 3027 (1:1,000 for immunoblots)
12. Mouse monoclonal anti-bata-actin (C4) : Santa Cruz, Cat# sc-47778 (1:1,000 for immunoblots)

Validation

All the antibodies were bought from commercial vendors and were validated by the correspondent manufacturer, which is described in the manufacturer's website. Our usage was described in the Methods section of the manuscript. Most antibodies used are standard in the field and are cited in the manuscript where relevant :
1. Use of rabbit monoclonal anti-Myc-Tag (CST) for immunoblot was validated in whole cell lysate of transfected HEK293T in Zhang et al., Nature 553.7686 (2018): 91-95.
2. Use of rabbit polyclonal anti-Strep tag II (abcam) for immunoblot was validated in whole cell lysate of transfected HEK293T in Berrout et al., Nature communications 8.1 (2017): 1-15.
3. Use of mouse monoclonal anti-His tag (Genescript) for immunoblot was validated in pull-down assay using recombinant proteins in Hiyama et al., Nature communications 8.1 (2017): 1-13.
4. Use of rabbit monoclonal anti-IGFBP3 (CST) for immunoblot was validated in whole cell lysate of human NPC cells in Yin et al., Cell death & disease 11.7 (2020): 1-15.
5. Use of mouse monoclonal anti-IGFBP3 (Santa Cruz) for immunoblot was validated in co-IP using recombinant protein and medium of human A549 cell in Muterspaugh et al., Biochemistry 57.39 (2018): 5726-5737.
6. Use of mouse monoclonal anti-ALS (Santa Cruz) for immunoblot was validated by Santa Cruz in whole cell lystate of transfected HEK293T at a dilution of 1:100.
7. Use of mouse monoclonal anti-IGF1 (Thermo Fisher) for immunoblot was validated in photo-crosslinking assay using recombinant proteins in Pompach et al., Frontiers in endocrinology 10 (2019): 695.
8. Use of rabbit monoclonal anti-Phospho-IGF1 Receptor beta (Tyr1135/1136) (CST) and rabbit ponoclonal anti-IGF1 Receptor beta (CST) for immunoblot was validated in whole cell lysate of human NCI-H1975 cells in Yamaguchi et al., Nature communications 7.1 (2016): 1-13.
9. Use of mouse monoclonal anti-bata-actin (Santa Cruz) for immunoblot was validated by Santa Cruz in whole cell lysates of HeLa, Jurkat, K-562, and HEK293T cells at a dilution of 1:200.

Eukaryotic cell lines

Policy information about cell lines

Cell line source(s)

1. Expi293F cells: Thermo Fisher, Cat# A14527
2. HEK293A cells: Thermo Fisher, Cat# R70507

Authentication

The cell lines were not authenticated since they were purchased commercially and not commonly misidentified.

Mycoplasma contamination

The cell lines were not tested for mycoplasma contamination, because we immediately used after purchase from manufactures.

Commonly misidentified lines
(See ICLAC register)

No commonly misidentified cell lines were used.
